# Machine Learning Techniques Based on Primary User Emulation Detection in Mobile Cognitive Radio Networks

**DOI:** 10.3390/s22134659

**Published:** 2022-06-21

**Authors:** Ernesto Cadena Muñoz, Luis Fernando Pedraza, Cesar Augusto Hernández

**Affiliations:** Technological Faculty, Universidad Distrital Francisco José de Caldas, Bogotá 111931, Colombia; lfpedrazam@udistrital.edu.co (L.F.P.); cahernandezs@udistrital.edu.co (C.A.H.)

**Keywords:** mobile cognitive radio network, spectrum sensing, software-defined radio, primary user emulation

## Abstract

Mobile cognitive radio networks (MCRNs) have arisen as an alternative mobile communication because of the spectrum scarcity in actual mobile technologies such as 4G and 5G networks. MCRN uses the spectral holes of a primary user (PU) to transmit its signals. It is essential to detect the use of a radio spectrum frequency, which is where the spectrum sensing is used to detect the PU presence and avoid interferences. In this part of cognitive radio, a third user can affect the network by making an attack called primary user emulation (PUE), which can mimic the PU signal and obtain access to the frequency. In this paper, we applied machine learning techniques to the classification process. A support vector machine (SVM), random forest, and K-nearest neighbors (KNN) were used to detect the PUE in simulation and emulation experiments implemented on a software-defined radio (SDR) testbed, showing that the SVM technique detected the PUE and increased the probability of detection by 8% above the energy detector in low values of signal-to-noise ratio (SNR), being 5% above the KNN and random forest techniques in the experiments.

## 1. Introduction

The convergence services in the actual market increase the demand for more frequent resources of the radioelectric spectrum due to emerging new technologies. The methods for spectrum allocation lead us to inefficient use of the spectrum, and the frequency bands are assigned to a specific application to primary licensed users [1]. The mobile cognitive radio network (MCRN) seems to be an alternative solution for better spectrum utilization by using the spectrum holes to transmit the secondary signals, making better use of the spectrum [2].

The first process in the MCRN is to sense the spectrum to guarantee that the spectrum will be used for the secondary user (SU) just in the case that the primary user (PU) is not using it [3]. Spectrum sensing techniques send the sensing information to the detection algorithm to obtain a decision about the PU presence. One of the most used techniques is energy detection, a faster method to detect signals damaged by Gaussian noise [4] that does not need previous knowledge of the signal; this is important because modulation techniques are changing constantly in MCRN [5].

In this spectrum sensing process, a common type of attack that affects the performance of the MCRN is the primary user emulation (PUE). In this attack, a PUE attempts to mimic a PU transmission to obtain access to a specific frequency band by injecting a false PU signal in the specific frequency selected to make other SUs release their spectrum frequency [6]. The impact of PUE is high because it causes interference to the primary and cognitive network, underutilization of the spectrum, denial of service, and user disconnection secondary, among other factors [7].

In MCRN, the PU is the licensed user of the 4G/5G network, and the operator must secure the PU connection when it is needed. The essence of the MCRN is not interfering with the PU signal, which is why the cognitive system detects the presence of the PU to avoid the use of its frequency [3]. On the other hand, the PUE attacker is a malicious SU that imitates the PU signal and characteristics so that the MCRN recognizes it as a PU and obtains access to the frequency channel and prevents other SU from using the channel. PUE detection takes relevance to avoid PU interference and to identify a real SU of the MCRN [8].

One way used to detect the PUE is to estimate the modulation type of the received signal. The system throws a learning process to classify a PU as a signal that has some modulation type, for example, binary phase shift keying (BPSK). If the modulation is different, the signal is marked as PUE [9]. If the signal is based on 4G and 5G networks, several modulation schemes can be used, such as time division multiple access (TDMA) or orthogonal frequency division multiple access (OFDMA), and with the knowledge of its patterns, detection systems can recognize them. However, in an MCRN, the signals are random variables that change over time, which is why detection systems based on prior information, such as modulation type, can increase the probability of false alarm probability and decrease the probability of PUE detection [10].

Some authors translate the signals and analyze them in the frequency domain. With these changes, the entropy can be used to detect PU. Another advantage is that the detectors are less sensitive to noise, and the probability of detection increases. Some authors have used Shannon entropy to detect these signals, and it shows better results in terms of lower SNR signals [10,11].

In the literature, several PUE detection techniques have been proposed that are based on localization, position, statistical analysis, and physical layer variables. In techniques that try to find the localization or the position of the users, the transmission properties are used to identify them, for example, by using the received signal strength (RSS) indicator that shows us the received power. Another statistical approach is used to find patterns in the signal characteristics. The received signal can be used to extract some characteristics as an input for the system, including the RSS, time of the spectrum occupancy, and the interframe time, among others [6].

As a complement, machine learning techniques have been used for telecommunication applications in areas such as security, data classification, and outlier detection. In [12], the authors use a decision tree and SVM to detect an attack called denial of service in emergent networks such as the software-defined network (SDN). In [13], the authors show the potential of machine learning to increase the performance of the intrusion detection system by using a classification method based on judgment feedback. In [14], machine learning was used to detect outliers in the Internet of Things and wireless sensor networks, showing the possibilities and versatility of these techniques.

In this paper, we propose using machine learning techniques such as random forest, SVM, and KNN to detect PUEs in MCRNs by using a previous learning process that allows us to establish a database of parameters such as the SNR and Shannon entropy of the received power signal. In this method, there is no need to recognize the modulation type or any RF characteristics a priori. We also implemented the system in SDR devices, allowing us to obtain experimental results of the MCRN with real phones connected to the network, and a PUE implemented in another SDR, allowing us to contrast the theory and simulations of the network environment. The combination of energy and entropy detectors increases the probability of detection of the PUE in a lower SNR with a low increase in the computational complexity and detection time.

The implemented machine learning techniques are used to classify and detect whether the received signal at a specific frequency comes from a PU or an SU identifying the PUE with a high probability of detection above 90%. This solution increases the probability of detection of a single energy or entropy detection system and shows us the results of the three methods for MCRN signals in theory, simulation, and the SDR environment.

The paper is organized as follows. Section 2 illustrates the previous work and contributions; Section 3, the machine learning detection scheme; Section 4 focuses on experiments in SDR; Section 5 shows the results and discussions; and Section 6 shows the conclusions.

## 2. Previous Work

Spectrum sensing is the main feature of the MCRN, and it helps to avoid interference between the PU and SU [15]. It is found in the literature that the most commonly used technique for spectrum sensing in cognitive radio networks (CRNs) is energy detection because its computational requirements are low. The power signal of the sensor center at a specific frequency is measured, and then the energy is calculated. Due to the noise of the environment and several SNR values, it is important to define or calculate a threshold that allows the detector to decide if a signal is noise or PU [16].

If we analyze that the PU is in movement in the MCRN, the SU power is changing according to its relative position from the PU. One way is to use dynamic-matching-based spectrum detection (DMBSD), which works better with a dynamic threshold and can sense data and make final sensing results, allowing an increase in the probability of detection while decreasing the false alarm probability by using a dynamic threshold and dynamic matching correlation-based sensing [17].

Another technique is cyclostationary feature detection (CFD), where the primary signal is filtered by finding the periodicity in the power signal. Signals such as sine waves are the carriers in the modulated signals, and the noise signal is not periodic, so it can be distinguished from the PU signal. The spectral and cyclic correlation functions are used instead of the power spectral density for finding PU. It is also compared with a fixed threshold to detect the PU presence but requires previous information on the transmitted signals [18].

Matched filter detection (MFD) is also used to find the PU presence in an additive white Gaussian noise (AWGN) channel, where it previously knows the parameters of a transmitted signal. The PU signal is passed through a filter, and a throw coherent method correlates the signal with the response of the matched filter. It requires knowing the modulation type and other information for the measurement [18].

In general, the spectrum sensing techniques used for PUE detection, can be divided in centralized and distributed, depending on the number of sensors available and the MCRN architecture [19]. Distributed schemes do not need a central entity; each node sends its sensing information to the sensors around it to make a decision based on the information received and its detection. It only needs to send information to its neighbors, and the energy consumption of the network is decreased in comparison to the centralized methods, which use a fusion center to make the decision [16]. As an example, the improvement of the performance in energy detection for a multiantenna scheme in a cognitive radio network has been explored with low SNR values and sensor-based cooperative spectrum sensing (CSS) in fading channels [4].

Techniques such as hard and soft fusion have been used for energy-efficient CSSs [20], and energy resources are very important if the SU in the MCRN is powered by a battery and needs to manage the energy efficiently. In these techniques, we can use AND, OR, and majority rules, and depending on the sensors, majority fusion shows better results [20].

In a CRN based on the infrastructure, a centralized scheme can be used with a central controller that connects all the SU, which is called the fusion center (FC). MCRN works in this scheme because the base station works like the FC by enabling SU communication. A dynamic spectrum allocation is proposed in [21]. An adaptive threshold works with the energy detector using methods for multiple attribute decisions, which maintains the QoS of the service even after handoff and performs better than the conventional fixed threshold [21].

Several approaches have been developed for detecting PUE. Conventional approaches include algorithms based on cryptography but then need to use key management, and the key distribution is not easy in a wireless environment. To solve this problem, an energy detection (ED) algorithm is proposed. Another solution uses a Markov random field combined with belief propagation for PUE detection, but its dependence on the ED increases the levels of false alarm. The physical layer is also exploited in some techniques, but it is open the research to find robust and intelligent algorithms to implement in these networks [22].

Currently, machine learning (ML) techniques are used to detect PUE attacks. The process is divided into two stages. The first stage is to train a machine with the data available, and once it is trained, it can classify the variables on the basis of the received signals. SVM is an option and has been used as a classification process [22]. In [23], the authors implement an SVM technique based on the SNR and the calculated Rényi entropy of the energy signal, and the results show that there is an improvement in the detection compared with ED for low SNR values. It starts with signal conditioning and filtering; this information is recorded for the learning process, which recognizes a PU in some modulations for several SNR values. After this, the classification process is made in real time to find the PUE.

Deep learning is also an option that uses artificial neural networks with an input such as an ED or cyclostationary signal. ED is used to recognize the PU presence, and after that, the cyclostationary is used to classify if there is an attack in the network. Another approach is to use the radiofrequency fingerprint by creating a profile for each transmitter that allows separation of the attack from a PU. Patterns of sparse coding have been used for the classification process in ML techniques [22]. The results show a high probability of detection, but it has a high computational complexity for the calculation of the neural network [22].

Another approach uses a secure sensing algorithm, which incorporates unsupervised machine learning to separate the PU signal from the PUE signal. It starts by identifying the licensed PU, and it is saved in the FC report that is shared with all the users connected to it. However, the false sensing reports caused by the PU have been identified thanks to the sensing history recorded in its database. Each user in the network uses a value to identify it in the FC. The reliability is then calculated, and the attackers can be classified and marked in the FC and shared with all the users. The problem of this solution is that a central unit is needed and its coverage is fixed, but in an MCRN, users are in movement, which leads to a hidden node problem in PUE attack detection [24].

Another implementation of ML for PUE identification is found in [6], which uses the Pattern-Described Link-Signature (PDLS) method to identify the features of the signals to classify the PU or SU in modulations such as Orthogonal Frequency Division Multiplexing (OFDM), but it depends on the modulation type than in an MCRN is difficult to predict due to its random nature [6].

In [25], the authors use machine learning in a wireless channel by using the locally weighted linear regression algorithm (LWLR) and predicting the available channels in a cycle and estimating the error distribution. With this information, a PUE attacker probability is estimated with an error threshold. This work can be used to estimate the probability of a PUE attack with a CRN, but it does not analyze a mobile user or a mobile network and is not implemented in an SDR device.

In [8], a time-distance with signal strength evaluation (TDSE) using an algorithm called extreme machine learning (EML) is used to prevent PUE attacks, and there are results with a variable number of PUE attackers and using some location-based techniques. This solution can be achieved by an MCRN, but in practice, the received values of the received signal strength are not stable and must be averaged; it also needs a fixed location PU and some devices to measure the power at different distances to identify the PUE attacker. In a real MCRN scenario, the PU, SU, and PUE are in movement, and there is not always a possibility of communication between them because of the limited coverage.

Another approach is to use a deep learning convolution network considering a fourth-order cyclic cumulant vector (CRM) in the frequency domain to detect the modulation patterns in a convolutional deep learning method. It uses an ideal PUE attacker with a limited functionality, and its analysis is for a fixed network using simulations and is not implemented in a real scenario with motional users [26].

Finally, to work with lower SNR values, the author in [11] proposes the analysis of the signals in the frequency domain with Gaussian minimum shift keying (GMSK) and OFDM, using the Rényi–Entropy method to classify whether the signal received is noise or the PU signal, which does not require previous knowledge of the signal patterns or modulation. It uses an SDR, and the results show that this technique has a better performance than the ED [11].

## 3. Machine Learning Techniques for PUE Detection in MCRN

**Figure 1:**
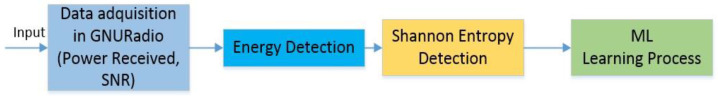
Proposed mixed entropy and energy detection for spectrum sensing.

The proposed model starts by acquiring some data in GNURadio, such as the power received and the SNR value in a specific frequency for testing, by using the SDR testbed that is described in Section 4. These values are used as inputs for the whole machine learning system, as shown in Figure 1. For each SNR value, 400 samples are taken to create a dataset for the learning and testing process.

The next step is to use energy and entropy detection algorithms with the previously collected data to generate a final entropy calculation for each SNR value, which will be used as the input for the learning process of the machine learning algorithms. The decisions are based on three inputs, the SNR value and entropy of the signal, and if there is an active PUE. As we activate the PUE in the SDR, the third value is known and recorded in the database.

The ML learning process starts by taking the three variable samples and splitting 75% for the training process and 25% for the testing process. The energy and the Shannon entropy are calculated, and with these values, the system is trained and ready to be probed.

After that process, we probe the results of the classification algorithms by calculating the PUE detection with the remaining values (25%) and comparing them with the real results for several SNR values, obtaining the probability of detection as the number of correct PUE detections over the number of testing samples.

It is important to define that in our solution, if the detectors decide that there is a PU/PUE present, the system changes its frequency to another one, preventing that PU from being affected. The first part of the general scheme can be seen in Figure 1, and the detectors and ML algorithms are defined below.

**Figure 2:**
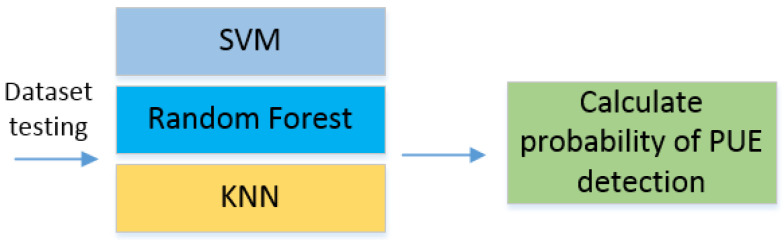
Machine learning techniques applied for PUE detection.

Once the learning and testing process are finished, we start to implement the different ML algorithms. For this paper, we used the SVM, random forest, and K-nearest neighbors (KNN). Finally, we probed the algorithms with the implementation in SDR and measured the probability of detection for PUE in MCRN as the number of correctly classified PUEs over the whole samples. This process can be seen in Figure 2. We selected a technique and measure the probability of detection using the dataset for testing with new data.

### 3.1. Energy Detection

The signal is measured by using GNURadio [27] connected to an SDR (USRP NI-2922). This signal is passed for a bandpass filter and compared with a threshold; for this case, the threshold is defined after a learning process to decide its value. An FFT is calculated, and the results are averaged. Then, the energy of the input is compared with the estimated threshold. If the measures are above the threshold, PU/PUE is present. The input signal is a PU that is generated by a cellular phone transmitting in the absolute radio frequency channel number (ARFCN) 226, which is centered at 888.8 MHz in DL [23].

A hypothesis test allows us to estimate the existence of a signal different from noise. By itself, it cannot distinguish a PU from a PUE signal, but it helps us to separate it from noise. In this binary test, *H*_0_ is identified as noise, and *H*_1_ represents that a PU/PUE signal is present with noise [15]:

(1) $$x(n)=\{\begin{array}{ccc} q(n) & & H_{0}\\ r(n)+q(n) & & H_{1} \end{array}$$

where *x*(*n*)** represents the input signal samples taken at time *n*, *q*(*n*)** is the noise, and *r*(*n*)** is the PU/PUE signal [15]. An energy detector of the received signal is represented by *Z*, and the threshold (*λ*) is calculated as in [15], and they are compared. The probability of detection (*P_d_*) is where the received signal is over the threshold and indicates that a PU or PUE signal is present in the environment. The probability of false alarm (*P_fa_*) is where the received signal is over the threshold, but there is no PU/PUE active, and it is represented in (2) [15].

(2) $$\begin{array}{l} P_{d}=p(Z\geq \lambda |H_{1}),\\ P_{fa}=p(Z\geq \lambda |H_{0}). \end{array}$$

### 3.2. Shannon Entropy Detection

The received signal is changed to analyze it in the frequency domain by using the Fourier transform for discrete values (DFT) according to Equation (3) [28], obtaining

(3) $$\bar{X}(k)=\{\begin{array}{ccc} \bar{Q}(k) & & H_{0}\\ \bar{R}(k)+\bar{Q}(k) & & H_{1} \end{array}$$

where *X*(*k*), *R*(*k*), and *Q*(*k*) represent the complex spectrum, the PU/PUE signal, and the noise, respectively. As in energy detection, a hypothesis test such as in ED is also used; *H*_0_ indicates that there is not a PU/PUE signal present and *H*_1_ indicates that there is a PU/PUE signal as in [29]. The entropy is then calculated by using the histogram technique, and the probabilities are calculated in (4) for a specific quantity of bins called *L*.

(4) $$H_{L}(X)=-\sum_{i=1}^{L}p_{i}\times \log(p_{i})$$

where *p_i_* is the probability of occurrence in the *i*-th bin. To probe this hypothesis, a test statistic is used for *L*, and the Shannon entropy is then estimated in (5).

(5) $$T(X)=-\sum_{i=1}^{L}\frac{k_{i}}{N}\times \log\left(\frac{k_{i}}{N}\right)\{\begin{array}{c} \leq \lambda :decide\begin{array}{cc} & H_{1} \end{array}\\ >\lambda :decide\begin{array}{cc} & H_{0} \end{array} \end{array}$$

The threshold, in this case, is calculated by a specific *P_fa_* [28]; *k_i_* denotes the total number of events in the *i*-th bin, and a value of *L* = 15 is as in [30].

### 3.3. Machine Learning Techniques

In this section, we define each of the techniques used for PUE detection, support vector machine (SVM), random forest, and KNN.

#### 3.3.1. Support Vector Machine

An SVM identifies the optimal hyperplane to separate two signals in the dataset. Traditional SVM has been used in several problems of engineering, such as network security, data classification, and bioinformatics [31].

A linear kernel is used when the function tends to be linearly separable and uses small samples and the training process is short. Another kernel, such as a polynomial, can be used when the function is not linearly separable and uses variables such as the polynomial degree that can affect the computational time and results [31].

**Figure 3:**
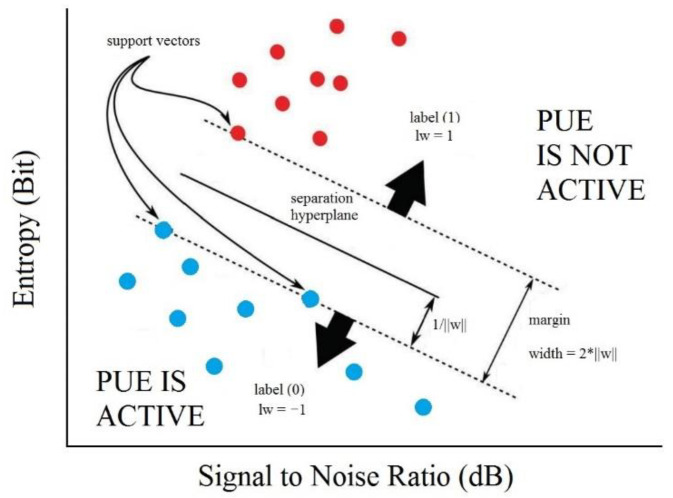
SVM operation with two dimensions; adapted from [32].

The SVM is a classification model that takes an input into a high-dimensional feature space, and depending on the data type, it uses kernel functions to separate it into two or three dimensions. The data points in the margin limits are the support vectors and help to calculate the optimized hyperplane; this process is shown in Figure 3 [32].

In Figure 3, *H* is the separating hyperplane. The separation margin is optimized to be the maximum distance of the support vectors of each category. The first objective is to separate the two parts of the data in this case linearly [33]. The second is to find the margin as in (6) [32], where *x* is the argument, *w* is the weight of the margin, and *b* is a constant.

(6) $$\begin{array}{l} \min\left(\frac{1}{2}{\left\|w\right\|}^{2}\right)\\ \begin{array}{cc} s.t. & \begin{array}{c} y_{i}(w\times x_{i}+b)\geq 1\\ i=1\ldots n \end{array} \end{array} \end{array}$$

To solve this optimization problem, a Lagrange dual method is implemented as applied in [33]. The functions are represented in (7).

(7) $$\begin{array}{l} f(x)=\mathrm{sgn}((w\times x_{i})+b)\\ f(x)=\mathrm{sgn}(\sum_{i=1}^{n}a_{i}\times y_{i}(x_{i}\times x+b)) \end{array}$$

where *x_i_* represents the support vectors, a is the Lagrange vector, and the symbolic function is *sgn*(*x*) [33].

**Figure 4:**
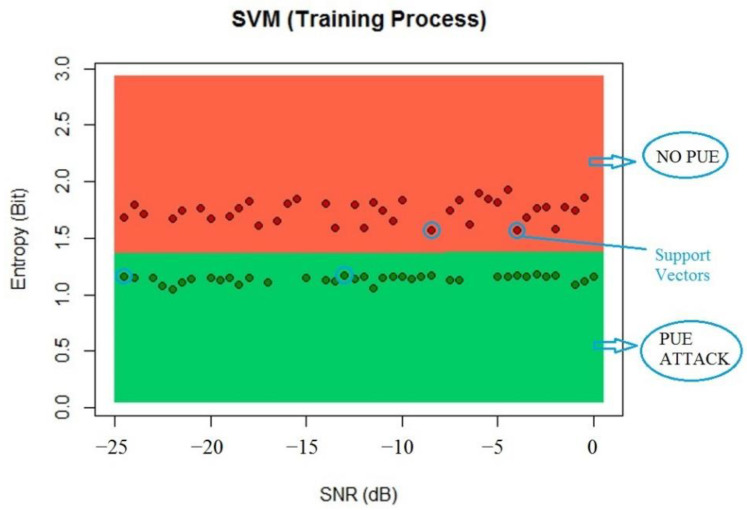
Example of SVM for PUE detection.

For our proposal, the input data for the SVM are the estimated entropy of the received signal and the measured SNR. The classification problem in this case is to detect if there is a PUE presence in the radio environment, which is achieved by comparing the entropy values and the SNR when PUE is on and when it is off. Then, we have three inputs to the system, the entropy in bits, the SNR in dB, and a binary variable that tells the system if there is a PUE attack. Then, the decision is made for each SNR value, and the supported vectors allow the linear kernel to separate the entropy for each SNR value. This algorithm maximizes the separation of the supported vectors, allowing better results than other techniques for PUE attack detection systems. The output of the system is the final decision that a PUE attack was detected in the radio environment at the selected frequency. An example of the application of the SVM for PUE detection can be seen in Figure 4.

#### 3.3.2. Random Forest

**Figure 5:**
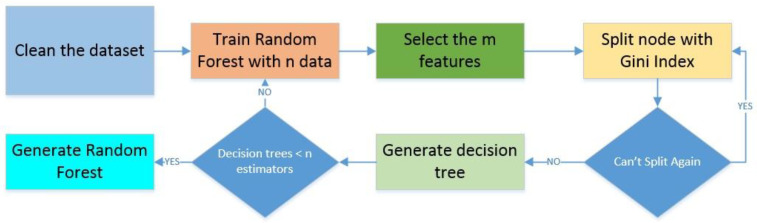
Random forest algorithm; adapted from [34].

The random forest technique is part of the supervised learning process. It is based on a simple decision tree, but in this case, many trees are created in multiple ways depending on the features and with random sampling for each tree. The final classification decision is based on the majority rule. It reduces the overfitting presented on a single tree and can calculate the main features of the dataset [34]. The process can be seen in Figure 5.

It has been implemented for classification and it shows better results than a simple tree and other techniques depending on the data composition and the dimensionality. Each tree takes values from a random sample using a unique distribution around all the trees in the forest. If there are too many trees, the error tends to a limit. We can use some algorithms to find the optimal number of trees for an estimated error and can be used to predict or classify data [35].

The main process is divided into two parts: the first is to calculate the decision trees individually, and the second is to apply the majority rule to classify the data. For this objective training, a set must be randomly selected, the random forest must be calculated, and the nodes must be split. In this last part, the Gini coefficient is calculated, and the feature with the lower value is selected as the split feature. The calculation of the coefficient is made according to [36].

1. Use Equation (8) to calculate the coefficient.

(8) $$\mathrm{Gini}(S)=1-\sum_{i=1}^{m}Pi^{2}$$

where *i* is the probability that category *j* is in dataset *S*.

2. Calculate the split node by using (9).

(9) $${\mathrm{Gini}}_{split}(S)=\frac{\left|S1\right|}{\left|S\right|}\mathrm{Gini}(S1)+\frac{\left|S2\right|}{\left|S\right|}\mathrm{Gini}(S2)$$

where |*S*| is the number of samples in the dataset and |*S*1| and |*S*2| represent the number of samples in the subsets [36].

After the definition of the technique, the input data for the random forest are the estimated entropy of the received signal and the measured SNR, which are the features for the training of the random forest, with the same parameters for the splitting of 75% for training and 25% for testing. We choose 10 trees for the random forest, as is analyzed in Section 4. The classification problem is to detect the PUE comparing the input parameters with the previous learning values and decide if there is a PUE present.

#### 3.3.3. K-Nearest Neighbors

The KNN technique is based on the distance between the sampling point of data and the extracted data points of the known neighbors. The classification decision is based on the K-nearest sampling points. The process consists of the distance calculation between the sampling points and the new data; then, the closest neighbors are estimated, and finally, a classification decision is made. As with other M techniques, data are divided into the training set with N samples and M features and the testing set that is used to probe the algorithm performance. K is the number of neighbors used in the calculation [37].

For the first step, there are several methods to calculate the distance between two data points (e.g., Euclidean distance and Manhattan distance, among others). The most common distance used in KNN is the Euclidean distance, which uses the sum of squared differences, as represented in (10) [37].

(10) $$d_{Euc}(A,B)=\sqrt{\sum_{i=0}^{M}{\left(A_{i}-B_{i}\right)}^{2}}$$

**Figure 6:**
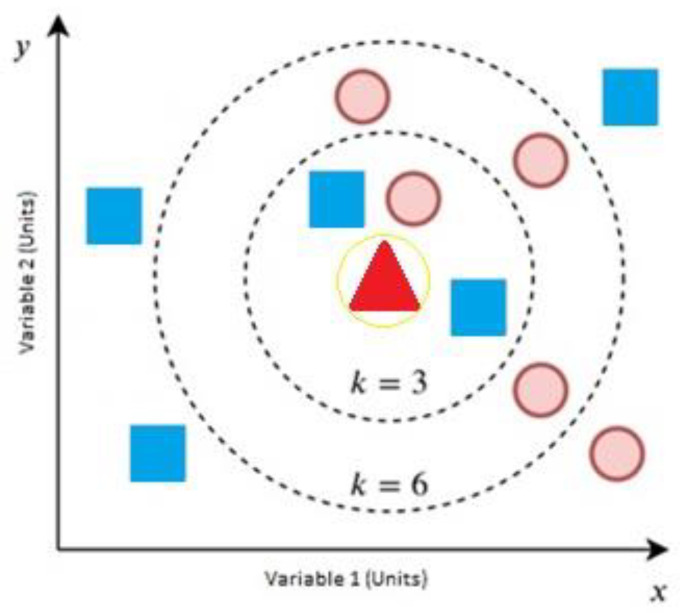
KNN decision example; adapted from [37].

Second, the distances are calculated and sorted, the K smallest values are extracted and selected for training data, and then the most common result in the samples is calculated [37]. An example of the KNN algorithm can be seen in Figure 6—it is used to classify an unknown bidimensional sample (green) by using the training set with two variables, blue and red. As an example, if we select k = 3, most of the neighbors are blue, so the final decision is that the new data belong to blue. If we change it to 6, the result shows that most of them are red, and we have to take care in the selection because if we select an even value for K and the results for the two classes are the same, the algorithm will not make a final decision [37].

For this case, we used the estimated entropy of the received signal and the measured SNR as the input variables, and for each SNR value, we chose five neighbors to make a stable decision. The result shows whether a PUE attack is present or not by analyzing whether the neighbors have been identified as active or not in the previous learning process.

## 4. Experiments

**Figure 7:**
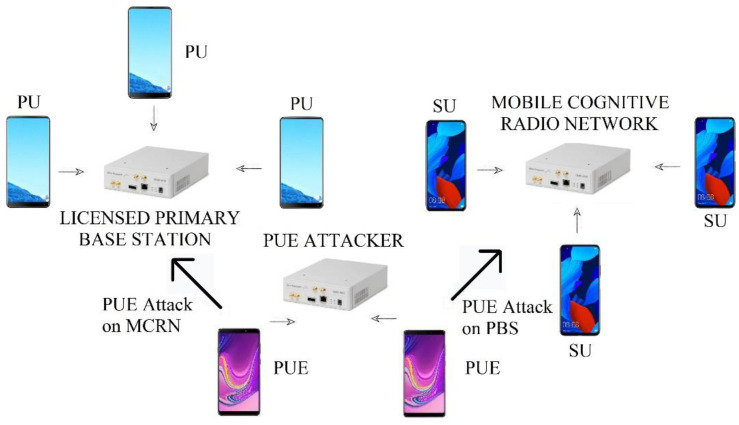
Testbed for SDR experiments.

We needed to capture the energy signal; for this purpose, we used an RTL-SDR 2832 device that is low-cost for spectrum analysis and is connected to a PC that works like an MCRN base station. For MCRN features, we used an NI USRP-2922 device with GNURadio software [27]; this device emulates an MCRN with frequency hopping. The PUE was implemented in another device with the same features, and it transmitted a malicious SU signal in the chosen frequency. The uplink (UL) signal was used to measure the energy. The testbed can be seen in Figure 7.

**Figure 8:**
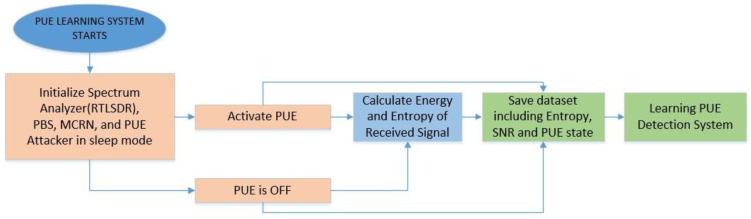
PUE learning system flowchart.

In general, we can divide the ML techniques into two parts: the learning and the classification process. For the learning process, we configured the devices to read the samples of a real phone call of a local operator. We calculated the energy, the received entropy, and the SNR. These values were used for the learning algorithm of the three techniques. The energy was averaged from the RTL-SDR signals received. Then, entropy and SNR were estimated two times, when there was an active PUE and in the absence of the PUE. The protocol was configured to detect a PU signal and release the channel to avoid interfering with this PU. This part of the process is shown in Figure 8.

RStudio [38] is the software used for the application of the ML techniques defined previously. The ML algorithms were implemented using class, e1071, random forest libraries of ML, and the dataset was continuously updated. Python programming was used to configure the base station and the PUE attack and OpenBTS software for the real phone call emulation [39]. Two midrange phones were used for the phone call, and all the algorithms were implemented on a PC working with Ubuntu 14.

**Figure 9:**
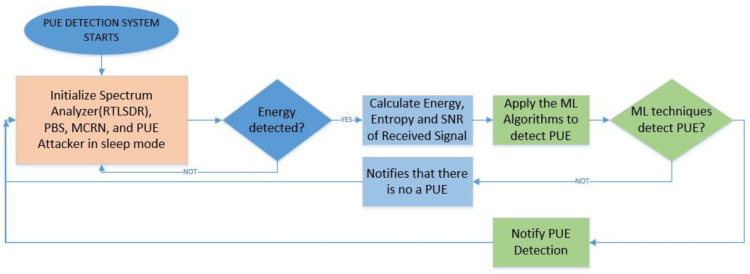
PUE detection system flowchart.

Once the learning process was completed, we continued with the detection of signals. For this process, each algorithm worked individually, and after a defined number of samples, it calculated whether there was a PUE attack present or not. We used the testing dataset to calculate the simulated probability of detection, and after all calculations, we estimated the detection with the real values when the PUE was active. This process can be seen in Figure 9.

The learning process was made only one time, and after that, we updated the dataset that was used for the ML algorithm processing, obtaining a final value for each algorithm that showed if there was a PUE attack present. We were able to make the algorithms work individually or make a complete solution that compares the values, and with an AND/OR logic, we obtained a final decision. The results are analyzed in the next section.

## 5. Results

In this section, the results of the test scenarios are presented and analyzed.

### 5.1. SDR Experiments

In the learning process, the stations were configured, and the authentication of the phones was started. The input of the systems was the UL signal from a phone call between two testing phones. The system calculated the SNR and energy of the input and calculated the entropy. This process was performed when the PUE attack was active and inactive, allowing the machine learning techniques to learn the behavior of the signals in time. We used a testing frequency that was in the 850 MHz range for MCRN. The received signal was captured by the RTL-SDR, and 100 samples were used to calculate one energy value. A total of 10000 samples were taken at different SNR values to allow the ML algorithms to recognize values with a good confidence level above 95% and with an estimated error lower than 5%.

**Figure 10:**
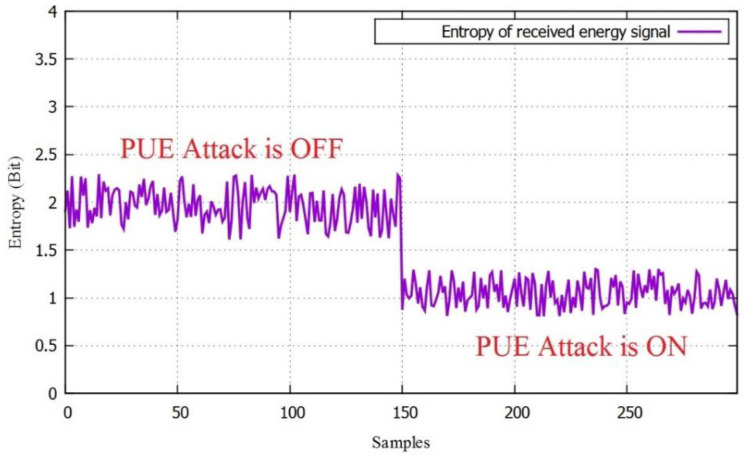
The entropy of the received energy signal.

With this energy, the entropy value was calculated for two scenarios. The first part was when there was just noise, and the second part was when a PUE attack was present. In this example, the first 150 samples were where there was just noise and PUE was not present, and the other 150 samples were taken when the PUE Attack was on. The entropy values can be seen in Figure 10 for a −10 dB SNR value.

We started the learning process by taking 400 samples of entropy for each SNR value from −25 dB to 0 dB in the SDR working as an MCRN. The parameters of the experiment can be seen in Table 1. We found the service, frequency, samples, confidence level, and estimated error. A total of 10,000 samples were calculated. For the experiment, we took 75% for the learning process and 25% for testing the ML algorithms.

After the calculation of these parameters, a random process split the dataset by taking 75% of the data for learning and 25% for testing the algorithms. The experiment was divided into three algorithms to obtain the results. The dataset was cleaned of null, empty, or outlier values before processing, and values were scaled to start the algorithms if needed. The results for the three algorithms were divided into two parts. The first one was the result of the training process with 75% of the samples in several SNR values from −25 dB to 0 dB. The second part described the testing process achieved with 25% of the original dataset, and with these results values, a theoretical probability of detection of the PUE was calculated.

#### 5.1.1. SVM Algorithm Results

**Figure 11:**
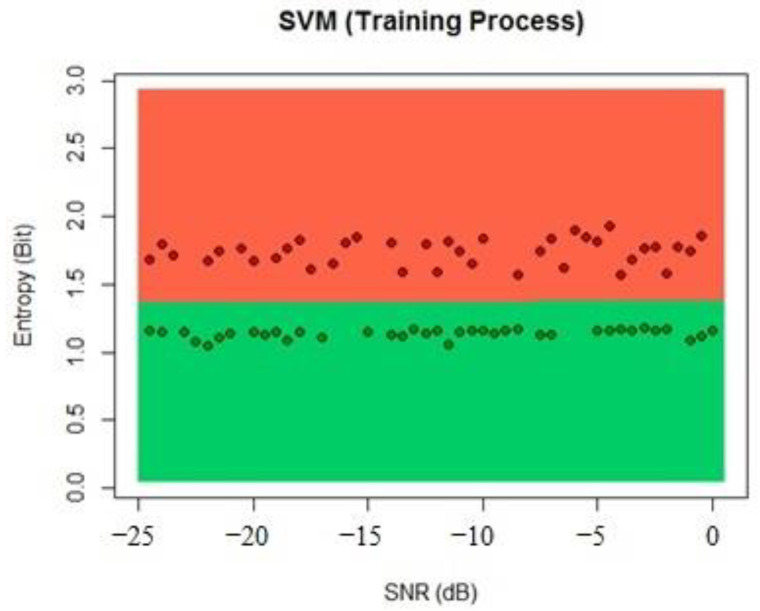
SVM training process classification.

For the SVM algorithm, we inserted the dataset as a data frame and called the classifier from library e1071 using C-classification and a linear kernel because the previous results obtained can be linearly classified and the number of support vectors was five, according to the algorithm. The results of the training process can be seen in Figure 11.

**Figure 12:**
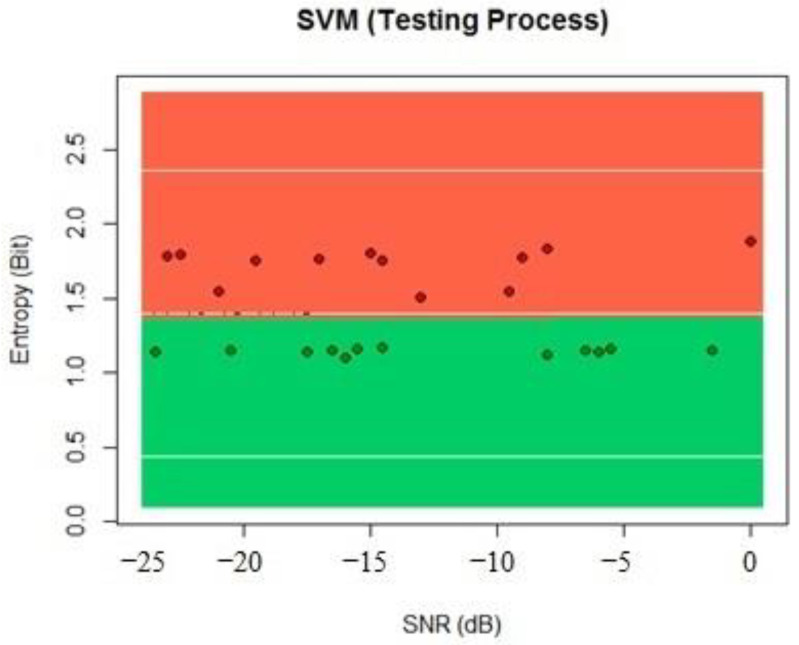
SVM testing process classification.

The entropy values worked with lower SNR values and helped the algorithm to obtain better results than energy by itself with the SVM. We processed the testing data to compare the results of the algorithm, which showed a classification of 99% of the results with this dataset, as seen in Figure 12.

#### 5.1.2. Random Forest Algorithm Results

**Figure 13:**
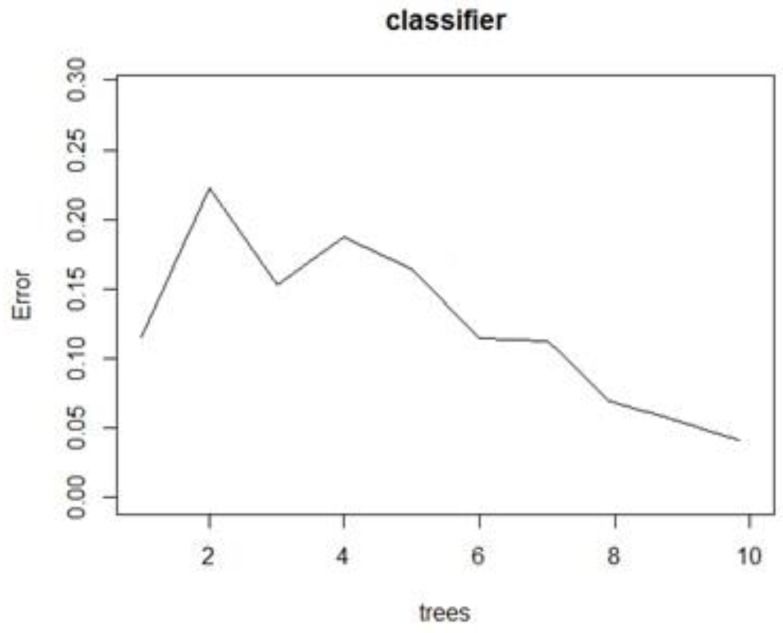
Estimated error vs. number of trees in the random forest algorithm.

The same split process was achieved by the three ML algorithms for the training and testing datasets. In a random forest, we chose a value of 10 for the number of trees with an estimated error lower than 5%, as seen in the black line of Figure 13. These values were estimated by the random forest algorithm, and our interest was to prevent overfitting.

**Figure 14:**
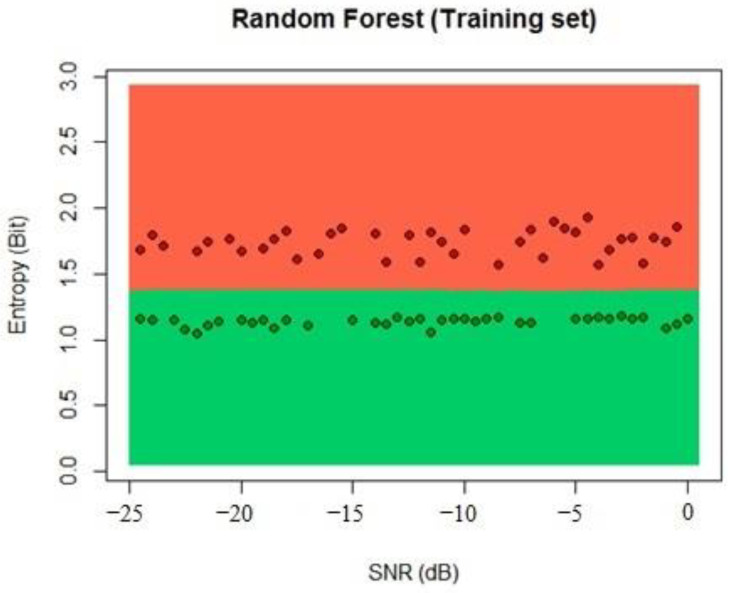
Random forest training process classification.

Then, with the 10 trees, the algorithm classified the training set when there was a PUE active and not, and the results of this process with the random forest function of the random forest library of R can be seen in Figure 14.

**Figure 15:**
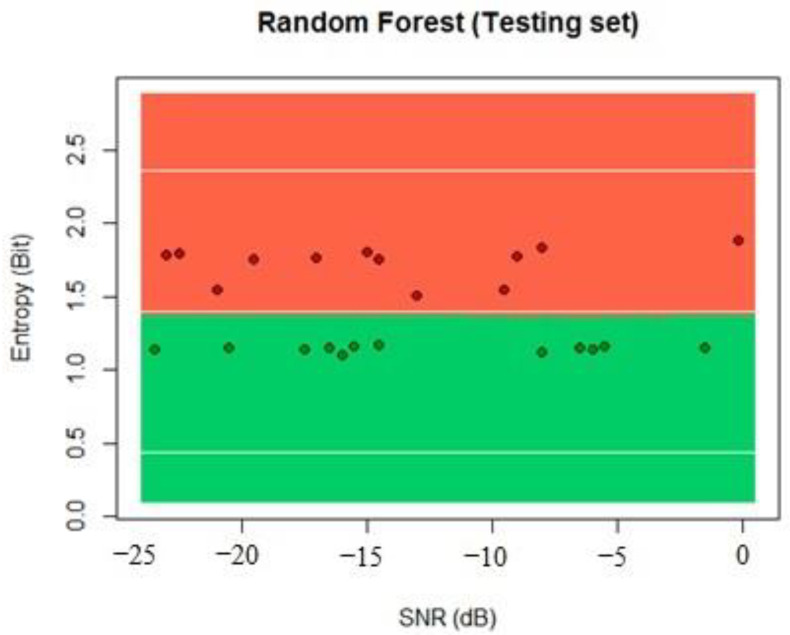
Random forest testing process classification.

We proceeded to calculate the results with the testing dataset, and the results showed that 99% of classification samples were classified correctly because good linear separation can be achieved by using the entropy of the received signal, as seen in Figure 15.

#### 5.1.3. KNN Algorithm Results

**Figure 16:**
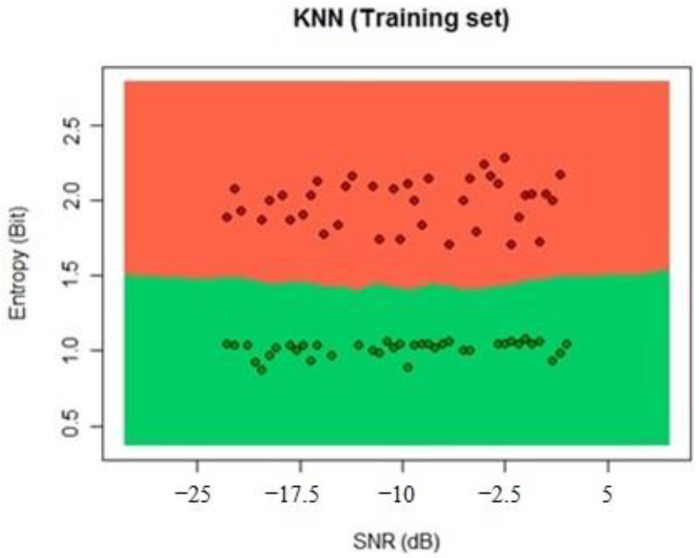
KNN training process classification.

For the KNN algorithm, five neighbors were chosen to prevent errors in equal decisions of the classification process. We used the KNN algorithm from the library class, and with the selection of the neighbor’s number, we proceeded to the training process that can be seen in Figure 16.

**Figure 17:**
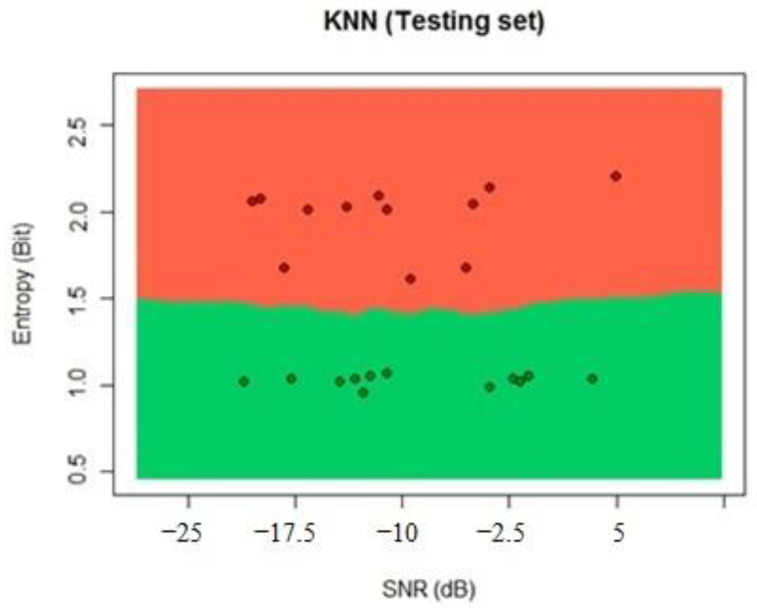
Random forest testing process classification.

We calculated the results of the KNN algorithm by using the testing dataset; according to these results, 99% of the samples were classified correctly. The KNN classification for these data can be seen in Figure 17.

After the three algorithms were calculated and the results of simulations were taken in a dataset, we continued to probe the algorithm in real time with the PUE attack and extracted the results in the SDR environment in the next section as general algorithm results.

#### 5.1.4. General Algorithm Results

After training was conducted, the last part of the process was to calculate the PUE probability of detection (PD) in the SDR environment. For this purpose, the position of the PUE was variable for experimental purposes, and we located it in the positions corresponding to the SNR measures. One hundred samples of entropy were taken on each point and averaged, and then each value was processed by the three algorithms. An OR logic was implemented; if any of the three algorithms decided that there was a PUE presence, the system showed a warning, and with the data obtained, included the device in the blacklist of PUE attackers. The parameters of the experiments can be seen in Table 2.

**Figure 18:**
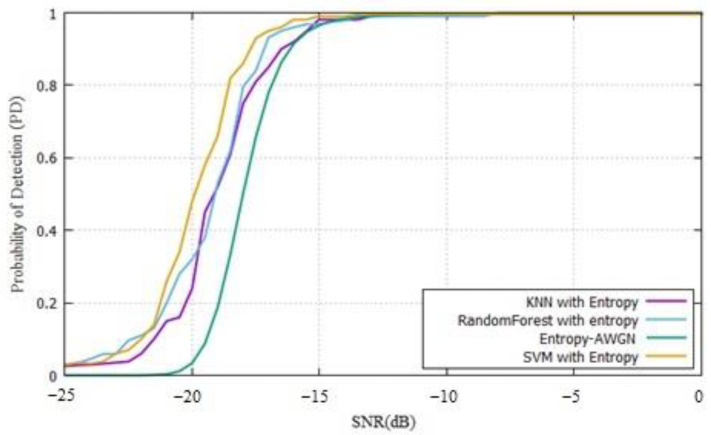
Probability of detection with ML techniques compared to entropy from [23].

The PD of PUE is calculated as the number of real PUE detections over the times that PUE is present. For this case, we measured with steps of 1 dB, and the results can be seen in Figure 18. These results can be compared with a previous entropy detection of PUE [23].

Finally, we measured the time response for each algorithm in one sample in the detection process, showing that the faster method was entropy detection and the SVM took more time, but any of the working methods protected the PU, allowing frequency hopping when a PU/PUE was detected while it evaluated if there was a PUE attack present. These results can be seen in Table 3 for one sample. These results showed that ML detection techniques can be optimized in time by using the KNN and random forest, and if we wanted to optimize the detection in lower SNR values, we could use the SVM.

## 6. Discussion

The obtained results using the ML techniques indicated that the proposed method increased the PD of PUE in real scenarios for MCRN, working better than a simple energy detector or even the entropy detector, compared with previous works [11,23]. The simulations used the same parameters found in the literature, and the results using the SDR showed that this solution worked even with low SNR values. A detection above 90% was found close to −18 dB, and with these results, the SVM worked better than other methods by 5%. The random forest and KNN results were very close and worked better than entropy by approximately 10%.

This solution worked better than energy detection because we did not have to measure or calculate a threshold and it was less vulnerable to noise. The three algorithms ran in a PC with Linux, and the computational complexity was medium in the first part of the learning process but low in the real-time detection because it made a comparison with the previous learning and classified the signal as a PUE attack or not. The fusion of the three techniques in OR logic worked better than individual approaches and allowed us to find a PD of 90% at −18 dB.

In the experiments, our solution worked better than the time arrival or localization methods because they were based on the received energy from a PU with a fixed location, but in the case of an MCRN, PU and SU were in movement, which was why these methods led to a low probability of detection. Due to the entropy calculation, our solution did not depend on a threshold and worked better in lower values of SNR and with users in movement. We emphasized that unlike other works, ours was implemented in SDR equipment and with real phone calls and PUE attacks to check the effectiveness of the detection and the system. After the learning process, our SDR testbed can detect PUE attacks very fast, protecting the licensed PU.

## 7. Conclusions

Machine learning techniques can be used to classify the presence of a PUE attack in an MCRN. Our work was probed in an indoor environment, and it achieved higher percentages of the probability of detection with a low complexity system that worked even with lower SNR values. The classification process obtained high values of classification because in this case, we worked with two features and they were linearly separated thanks to the use of entropy instead of energy for the PUE detection.

The results showed that the ML techniques were above the energy or entropy found in the literature, and an estimated PD value of 90% can be obtained at −18 dB. The energy detector was 12 dB above and 3 dB over the entropy detector. SVM worked 5% better than other ML techniques and KNN, and random forest worked better than entropy by 10% in terms of PD.

The experiments showed that an MCRN can be achieved in an SDR device, and with the modification of the source code, they can exploit the CRN capabilities. The PUE attack was replicated in this network, and with these techniques, it can be detected with high probability values, securing the PU networks and devices. Our solution worked better than energy, location, or entropy detection techniques in the literature, and it was implemented in SDR devices testing the detection time and the probability of detection with a phone call in MCRN.

## Figures and Tables

**Table 1:** Experimental parameters for the learning process.

Parameter	Value
Number of samples	10,000 samples
Averaged values	400 samples for each SNR value (−25 dB to 0 dB)
Noise signal	AWGN
Service	Phone Call-PUE
Frequency	831.8 MHz
Time of measurement	1 s for each SNR value
Confidence level	95%
Margin of error	5%

**Table 2:** Experimental parameters for the learning process.

Parameter	Value
Samples	2500 samples
Averaged values	100 samples for each SNR value (−25 dB to 0 dB)
Noise signal	AWGN
Service type	Phone Call-PUE
Frequency selected	831.8 MHz
Time of measurement	1 s for each SNR value
Confidence level	95%
Estimated error	5%

**Table 3:** Experiment results in algorithm detection time.

Entropy	KNN	Random Forest	SVM
100 ms	680 ms	750 ms	1000 ms

## References

[1] Verma G., Shukla S., Chauhan S.S. Analysis of Time-Bandwidth Product in Cooperative Spectrum Sensing in Cognitive Radio Network. Proceedings of the 2020 International Conference on Advances in Computing and Communication Engineering (ICACCE).

[2] Gao N., Jing X., Huang H., Mu J. (2017). Robust collaborative spectrum sensing using phy-layer fingerprints in mobile cognitive radio networks. IEEE Commun. Lett..

[3] Muñoz E.C., Rodriguez-Colina E., Pedraza L.F., Paez I.P. (2020). Detection of dynamic location primary user emulation on mobile cognitive radio networks using USRP. EURASIP J. Wirel. Commun. Netw..

[4] Zheng Y., Xia Y., Wang H. Spectrum Sensing Performance Based on Improved Energy Detector in Cognitive Radio Networks. Proceedings of the 2020 IEEE International Conference on Artificial Intelligence and Computer Applications (ICAICA).

[5] Thanuja T., Daman K.A., Patil A.S. Optimized Spectrum sensing Techniques for Enhanced Throughput in Cognitive Radio Network. Proceedings of the 2020 International Conference on Emerging Smart Computing and Informatics (ESCI).

[6] Albehadili A., Ali A., Jahan F., Javaid A.Y., Oluochy J., Devabhaktuniz V. Machine Learning-based Primary User Emulation Attack Detection In Cognitive Radio Networks using Pattern Described Link-Signature (PDLS). Proceedings of the 2019 Wireless Telecommunications Symposium (WTS).

[7] Muñoz E.C., Blanco H.J.E., Parra I.P.P. Detection of Malicious Primary User Emulation on Mobile Cognitive Radio Networks. Proceedings of the 2019 International Conference on Information Systems and Computer Science (INCISCOS).

[8] Sureka N., Gunaseelan K. (2021). Investigations on detection and prevention of primary user emulation attack in cognitive radio networks using extreme machine learning algorithm. J. Ambient Intell. Humaniz. Comput..

[9] Chakravarthy R., Huang K., Zhang L., Wu Z. Primary User authentication of cognitive radio network using underlay waveform. Proceedings of the 2017 Cognitive Communications for Aerospace Applications Workshop (CCAA).

[10] Nijsure Y., Kaddoum G., Ghodoosipour G., Cai G., Wang L. A novel spectrum sensing mechanism based on distribution discontinuity estimation within cognitive radio. Proceedings of the 2016 IEEE 84th Vehicular Technology Conference (VTC-Fall).

[11] Cadena Muñoz E., Pedraza Martínez L.F., Hernandez C.A. (2020). Rényi Entropy-Based Spectrum Sensing in Mobile Cognitive Radio Networks Using Software Defined Radio. Entropy.

[12] Sudar K.M., Beulah M., Deepalakshmi P., Nagaraj P., Chinnasamy P. Detection of Distributed Denial of Service Attacks in SDN using Machine learning techniques. Proceedings of the 2021 International Conference on Computer Communication and Informatics (ICCCI).

[13] He Y. Research on the Key Technology of Network Security Based on Machine Learning. Proceedings of the 2021 6th International Conference on Intelligent Computing and Signal Processing (ICSP).

[14] Ghosh N., Maity K., Paul R., Maity S. Outlier detection in sensor data using machine learning techniques for IoT framework and wireless sensor networks: A brief study. Proceedings of the 2019 International Conference on Applied Machine Learning (ICAML).

[15] Salama G.M., Taha S.A. Cooperative spectrum sensing and hard decision rules for cognitive radio network. Proceedings of the 2020 3rd International Conference on Computer Applications & Information Security (ICCAIS).

[16] Hajihoseini A., Ghorashi S.A. (2017). Distributed spectrum sensing for cognitive radio sensor networks using diffusion adaptation. IEEE Sens. Lett..

[17] Gu Y., Pei Q., Li H. (2019). Dynamic matching-based spectrum detection in cognitive radio networks. China Commun..

[18] Nandini K., Hariprasad S. A Survey of Spectrum Sensing Mechanisms in Wireless Cognitive Radio Networks. Proceedings of the 2017 14th IEEE India Council International Conference (INDICON).

[19] Chavan A.S., Junnarkar A. Dynamic Spectrum Sensing Method For Mobile Cognitive Radio Ad Hoc Networks. Proceedings of the 2020 International Conference on Emerging Smart Computing and Informatics (ESCI).

[20] Sharma G., Sharma R. Performance comparison of hard and soft fusion Techniques for Energy Efficient CSS in Cognitive Radio. Proceedings of the 2018 International Conference on Advanced Computation and Telecommunication (ICACAT).

[21] Divya A., Nandakumar S. Adaptive threshold based spectrum sensing and spectrum handoff using MADM methods for voice and video services. Proceedings of the 2019 International Conference on Vision towards Emerging Trends in Communication and Networking (ViTECoN).

[22] Aygül M.A., Furqan H.M., Nazzal M., Arslan H. Deep learning-assisted detection of PUE and jamming attacks in cognitive radio systems. Proceedings of the 2020 IEEE 92nd Vehicular Technology Conference (VTC2020-Fall).

[23] Cadena Muñoz E., Pedraza Martínez L.F., Ortiz Triviño J.E. (2020). Detection of Malicious Primary User Emulation Based on a Support Vector Machine for a Mobile Cognitive Radio Network Using Software-Defined Radio. Electronics.

[24] Li Y., Peng Q. Achieving secure spectrum sensing in presence of malicious attacks utilizing unsupervised machine learning. Proceedings of the MILCOM 2016-2016 IEEE Military Communications Conference.

[25] Yao H., Zhu G., Yang Y. Primary User Emulation Detection in Wireless Networks with Machine Learning Approach. Proceedings of the 2021 8th International Conference on Automation and Logistics (ICAL).

[26] Srinivasan S., Shivakumar K., Mohammad M. (2019). Semi-supervised machine learning for primary user emulation attack detection and prevention through core-based analytics for cognitive radio networks. Int. J. Distrib. Sens. Netw..

[27] Ettus C. (2019). Building and Installing the USRP Open Source Toolchain (UHD and GNU Radio) on Linux. https://kb.ettus.com/Building_and_Installing_the_USRP_Open-Source_Toolchain_(UHD_and_GNU_Radio)_on_Linux.

[28] Zhang Y.L., Zhang Q.Y., Melodia T. (2010). A frequency-domain entropy-based detector for robust spectrum sensing in cognitive radio networks. IEEE Commun. Lett..

[29] So J. (2015). Entropy-based Spectrum Sensing for Cognitive Radio Networks in the Presence of an Unauthorized Signal. KSII Trans. Internet Inf. Syst..

[30] Zhu W., Ma J., Faust O. (2013). A comparative study of different entropies for spectrum sensing techniques. Wirel. Pers. Commun..

[31] Mohan L., Pant J., Suyal P., Kumar A. Support Vector Machine Accuracy Improvement with Classification. Proceedings of the 2020 12th International Conference on Computational Intelligence and Communication Networks (CICN).

[32] Hoang T.M., Duong T.Q., Tuan H.D., Lambotharan S., Hanzo L. (2021). Physical layer security: Detection of active eavesdropping attacks by support vector machines. IEEE Access.

[33] Cheng G., Tong X. Fuzzy Clustering Multiple Kernel Support Vector Machine. Proceedings of the 2018 International Conference on Wavelet Analysis and Pattern Recognition (ICWAPR).

[34] Zhou Q., Lan W., Zhou Y., Mo G. Effectiveness Evaluation of Anti-bird Devices based on Random Forest Algorithm. Proceedings of the 2020 7th International Conference on Information, Cybernetics, and Computational Social Systems (ICCSS).

[35] Prihatno A.T., Nurcahyanto H., Jang Y.M. Predictive Maintenance of Relative Humidity Using Random Forest Method. Proceedings of the 2021 International Conference on Artificial Intelligence in Information and Communication (ICAIIC).

[36] Lan H., Pan Y. A crowdsourcing quality prediction model based on random forests. Proceedings of the 2019 IEEE/ACIS 18th International Conference on Computer and Information Science (ICIS).

[37] Vieira J., Duarte R.P., Neto H.C. (2019). kNN-STUFF: KNN streaming unit for Fpgas. IEEE Access.

[38] Allaire J. (2012). RStudio: Integrated development environment for R. Boston MA.

[39] Garcia J.H.H.A., Rodríguez C.J.P., Ruiz F.E.Ñ., Alarcón J.L.A. (2019). Implementación de una red celular GSM mediante software OPENBTS. PUEBLO Cont..

